# A Hybrid PAC Reinforcement Learning Algorithm for Human-Robot Interaction

**DOI:** 10.3389/frobt.2022.797213

**Published:** 2022-03-09

**Authors:** Ashkan Zehfroosh, Herbert G. Tanner

**Affiliations:** Cooperative Robotics Lab, Department of Mechanical Engineering, University of Delaware, Newark, DE, United States

**Keywords:** reinforcement learning, probably approximately correct, markov decision process, human-robot interaction, sample complexity

## Abstract

This paper offers a new hybrid probably approximately correct (PAC) reinforcement learning (RL) algorithm for Markov decision processes (MDPs) that intelligently maintains favorable features of both model-based and model-free methodologies. The designed algorithm, referred to as the Dyna-Delayed Q-learning (DDQ) algorithm, combines model-free Delayed Q-learning and model-based R-max algorithms while outperforming both in most cases. The paper includes a PAC analysis of the DDQ algorithm and a derivation of its sample complexity. Numerical results are provided to support the claim regarding the new algorithm’s sample efficiency compared to its parents as well as the best known PAC model-free and model-based algorithms in application. A real-world experimental implementation of DDQ in the context of pediatric motor rehabilitation facilitated by infant-robot interaction highlights the potential benefits of the reported method.

## 1 Introduction

While several reinforcement learning (RL) algorithms can apply to a dynamical system modeled as a Markov decision process (MDP), few are probably approximately correct (PAC)—meaning able to guarantee how soon the algorithm will converge to a near-optimal policy. Existing PAC MDP algorithms can be broadly divided into two groups: model-based algorithms like (Brafman and Tennenholtz, 2002; Kearns and Singh, 2002; Strehl and Littman, 2008; Szita and Szepesvári, 2010; Strehl et al., 2012; Lattimore and Hutter, 2014; Ortner, 2020), and model-free Delayed Q-learning algorithms (Strehl et al., 2006; Jin et al., 2018; Dong et al., 2019). Each group has its advantages and disadvantages. The goal here is to capture the advantages of both groups, while preserving PAC properties.

The property of an RL to have bounded regret is tightly closed to probable approximate correctness in the sense that it also provides some type of theoretical performance guarantee (Jin et al., 2018). Rl algorithms with bounded regret place a bound on the overall loss during the learning process, contrasting themselves to the case when the optimal policy is adopted throughout the whole process. Similar to PAC RL algorithms, existing RL algorithms with bounded regret are either model-based (Auer and Ortner, 2005; Ortner and Auer, 2007; Jaksch et al., 2010; Azar et al., 2017) or model-free (Jin et al., 2018), with none that are able to capture advantages of both groups. Interestingly, while all PAC RL algorithms also have bounded regret, the inverse is not always true (Jin et al., 2018).

Model-free RL is a powerful approach for learning complex tasks. For many real-world learning problems, however, the approach is taxing in terms of the size of the necessary body of data—what is more formally referred to as its *sample complexity*. The reason is that model-free RL ignores rich information from state transitions and only relies on the observed rewards for learning the optimal policy (Pong et al., 2018). A popular model-free PAC RL MDP algorithm is known as *Delayed Q-learning* (Strehl et al., 2006). The known upper-bound on the sample complexity of Delayed Q-learning suggests that it outperforms model-based alternatives only when the state-space size of the MDP is relatively large (Strehl et al., 2009).

Model-based RL, on the other hand, utilizes all information from state transitions to learn a model, and then uses that model to compute an optimal policy. The sample complexity of model-based RL algorithms are typically lower than that of model-free ones (Nagabandi et al., 2018); the trade-off comes in the form of computational effort and possible bias (Pong et al., 2018). A popular model-based PAC RL MDP algorithm is *R-max* (Brafman and Tennenholtz, 2002). The derived upper-bound for the sample complexity of the R-max algorithm (Kakade, 2003) suggests that this model-based algorithm shines from the viewpoint of sample efficiency when the size of the state/action space is relatively small. This efficiency assessment can typically be generalized to most model-based algorithms. Overall, R-max and Delayed Q-learning are incomparable in terms of their bound on the sample complexity. For instance, *for the same sample size*, R-max is bound to return a policy of higher accuracy compared to Delayed Q-learning, while the latter will converge much faster on problems with large state spaces.

Typically, model-free algorithms circumvent the model learning stage of the solution process, a move that by itself reduces complexity in problems of large size. In many applications, however, model learning is not the main complexity bottleneck. Neurophysiologically-inspired hypotheses (Lee et al., 2014) have suggested that the brain approach toward complex learning tasks can be model-free (trial and error) or model-based (deliberate planning and computation) or even a combination of both, depending on the amount and reliability of the available information. This intelligent combination is postulated to contribute to making the process efficient and fast. The design of the PAC MDP algorithm presented in this paper is motivated by such observations. Rather than strictly following one of the two prevailing directions, it orchestrates a marriage of a model-free (Delayed Q-learning) with a model-based (R-max) PAC algorithm, in order to give rise to a new PAC algorithm (Dyna-Delayed Q-learning (DDQ)) that combines the advantages of both.

The search for a connection between model-free and model-based RL algorithms begins with the Dyna-Q algorithm (Sutton, 1991), in which synthetic generated experiences based on the learned model are used to expedite Q-learning. Some other examples that continued along this thread of research are partial model back propagation (Heess et al., 2015); training a goal condition *Q* function (Parr et al., 2008; Sutton et al., 2011; Schaul et al., 2015; Andrychowicz et al., 2017); integrating an LQR-based algorithm into a model-free framework of path integral policy improvement (Chebotar et al., 2017); and analogies of model-based solutions for deriving adaptive model-free control law (Tutsoy et al., 2021). The recently introduced Temporal Difference Model (TDM) provides a smooth (er) transition from model-free to model-based, during the learning process (Pong et al., 2018). What is still missing in the literature, though, is a PAC combination of model-free and model-based frameworks.

In this paper, the idea behind Dyna-Q is leveraged to combine two popular PAC algorithms, one model-free and one model-based, into a new one named DDQ, which is not only PAC like its parents, but also inherits the best of both worlds: it will intelligently behave more like a model-free algorithm on large problems, and operate more like a model-based algorithm on problems that require high accuracy, being content with the smallest among the sample sizes required by its parents. Specifically, the sample complexity of DDQ, in the worst case, matches the minimum bound between that of R-max and Delayed Q-learning, and often outperforms both. Note that the DDQ algorithm is purely online and does not assume access to a generative model like in (Azar et al., 2013). While the provable worst case upper bound on the sample complexity of DDQ algorithm is higher than the best known model-based (Szita and Szepesvári, 2010) and model-free (Jin et al., 2018; Dong et al., 2019) algorithms, we can demonstrate (see Section 5) that the hybrid nature allows for superior performance of the DDQ algorithm in applications. The availability of a hybrid PAC algorithm like DDQ in hand obviates the choice between a model-free and a model-based approach.

The approach in this paper falls under the general category of *tabular reinforcement learning*, which basically encompasses problems where the state-space can admit a tabular representation. Outside this framework, namely in non-tabular reinforcement learning, one of the key advantages is the ability to handle really large state-spaces (Bellemare et al., 2016; Hollenstein et al., 2019), but this is not the particular focus of the approach here. Moreover, the emphasis here is on learning in MDPs with unknown but constant parameters (transition probabilities and/or reward function). This is also distinct from another thread of research that addresses uncertainty and robustness in MDPs whose parameters are randomly (or even adversely) selected from a set and can vary over the instances when the same state-action pair is encountered (Lim et al., 2013).

Our own motivation for developing of this new breed of RL algorithms comes from application problems in early pediatric motor rehabilitation, where robots can be used as smart toys to socially interact and engage with infants in play-based activity that involves gross motor activity. In this setting, MDP models can be constructed to abstractly capture the dynamics of the social interaction between infant and robot, and RL algorithms can guide the behavior of the robot as it interacts with the infant in order to achieve the maximum possible rehabilitation outcome—the latter possibly quantified by the overall length of infant displacement, or the frequency of infant motor transitions. Some early attempts at modeling such instances of human-robot interaction (HRI) did not result in models of particularly large state and action spaces, but were particularly complicated by the absence of sufficient data sets for learning (Zehfroosh et al., 2017; Zehfroosh et al., 2018). This is because every child is different, and the exposure of each particular infant to the smart robotic toys (during which HRI data can be collected) is usually limited to a few hours per month. There is a need, therefore, for reinforcement learning approaches that can maintain (or even guarantee) efficiency and accuracy even when the learning set is particularly small.

The paper starts with some technical preliminaries in Section 2. This section introduces the required properties of a PAC RL algorithm in the form of a well-known theorem. The DDQ algorithm is introduced in Section 3, with particular emphasis given on its update mechanism. Section 4 presents the mathematical analysis that leads the establishment of the algorithm’s PAC properties, and the analytic derivation of its sample complexity. Finally, Section 5 offers numerical data to support the theoretical sample complexity claims. The data indicate that DDQ outperforms its parent algorithms as well as the state-of-the-art model-base and model-free algorithms in terms of the required samples to learn near-optimal policy. Experimental results from application of DDQ in the context of early pediatric motor rehabilitation suggest the algorithm’s efficacy and its potential as part of a child-robot interface mechanism that involves autonomous and adaptive robot decision-making. To enhance this paper’s readability, the proofs of most of the technical lemmas supporting the proof of our main result are moved to the paper’s Appendix.

## 2 Technical Preliminaries

A finite MDP *M* is a tuple {*S*, *A*, *R*, *T*, *γ*} with elements

*S* a finite set of states
*A* a finite set of actions
*R*: *S* × *A* → [0, 1] the *reward* from executing *a* at *s*
*T*: *S* × *A* × *S* → [0, 1] the *transition probabilities*

$\gamma \in \left[0,1\right)$ the *discount factor*

A *policy* *π* is a mapping *π*: *S* → *A* that selects an action *a* to be executed at state *s*. A policy is *optimal* if it maximizes the expected sum of discounted rewards; if *t* indexes the current time step and *a*_*t*_, *s*_*t*_ denote current action and state, respectively, then this expected sum is written $E_{M}\left\{\sum_{t=0}^{\infty }\gamma^{t}R\left(s_{t},a_{t}\right)\right\}$ . The discount factor *γ* here reflects the preference of immediate rewards over future ones. The *value* of state *s* under policy *π* in MDP *M* is defined as

$$v_{M}^{\pi }\left(s\right)=E_{M}\left\{R\left(s,\pi \left(s\right)\right)+\overset{\infty }{\underset{t=1}{\sum }}\gamma^{t}R\left(s_{t},\pi \left(s_{t}\right)\right)\right\}$$

Note that an upper bound for the value at any state is $v_{\max}=\frac{1}{1-\gamma }$ . Similarly defined is the value of *state-action pair* (*s*, *a*) under policy *π*:

$$Q_{M}^{\pi }\left(s,a\right)=E_{M}\left\{R\left(s,a\right)+\overset{\infty }{\underset{t=1}{\sum }}\gamma^{t}R\left(s_{t},\pi \left(s_{t}\right)\right)\right\}$$ (1)

Every MDP *M* has at least one optimal policy π* that results in an optimal value (or state-action value) assignment at all states; the latter is denoted $v_{M}^{*}\left(s\right)$ (or $Q_{M}^{*}\left(s,a\right)$ , respectively).

The standard approach to finding the optimal values is through the search for a fix point of the Bellman equation

$$v_{M}^{*}\left(s\right)=\underset{a}{\max}\left\{R\left(s,a\right)+\gamma \underset{s^{\prime }}{\sum }T\left(s,s^{\prime },a\right)v_{M}^{*}\left(s^{\prime }\right)\right\}$$

which, after substituting $V_{M}^{*}\left(s^{\prime }\right)=\max_{a}Q_{M}^{*}\left(s^{\prime },a\right)$ , can equivalently be written in terms of state-action values

$$Q_{M}^{*}\left(s,a\right)=R\left(s,a\right)+\gamma \underset{s^{\prime }}{\sum }T\left(s,s^{\prime },a\right)v_{M}^{*}\left(s^{\prime }\right)$$

Reinforcement learning (RL) is a procedure to obtain an optimal policy in an MDP, when the actual transition probabilities and/or reward function are not known. The procedure involves exploration of the MDP model. An RL algorithm usually maintains a table of state-action pair value estimates *Q* (*s*, *a*) that are updated based on the exploration data. We denote *Q*_*t*_ (*s*, *a*) the currently stored value for state-action pair (*s*, *a*) at timestep *t* during the execution of an RL algorithm. Consequently, *v*_*t*_(*s*) = max_*a*_ *Q*_*t*_ (*s*, *a*). An RL algorithm is *greedy* if it at any timestep *t*, it always executes action *a*_*t*_ = arg max_*a*∈*A*_ *Q*_*t*_ (*s*_*t*_, *a*). The policy in force at time step *t* is similarly denoted *π*_*t*_. In what follows, we denote |*S*| the cardinality of a set *S*.

Reinforcement learning algorithms have been classified as *model-based* or *model-free*. Although the characterization is debatable, what is meant by calling an RL algorithm “model-based,” is that *T* and/or *R* are estimated based on online observations (exploration data), and the resulting estimated model subsequently informs the computation of the optimal policy. A model-free RL algorithm, on the other hand, would skip the construction of an estimated MDP model, and search directly for an optimal policy over the policy space. An RL algorithm is expected to converge to the optimal policy, practically reporting a near-optimal one at termination.

Probably approximately correct (PAC) analysis of RL algorithms deals with the question of how fast an RL algorithm converges to a near-optimal policy. An RL algorithm is PAC if there exists a probabilistic bound on the number of exploration steps that the algorithm can take before converging to a near-optimal policy.

**Algorithm 1:** The ddq Algorithm.

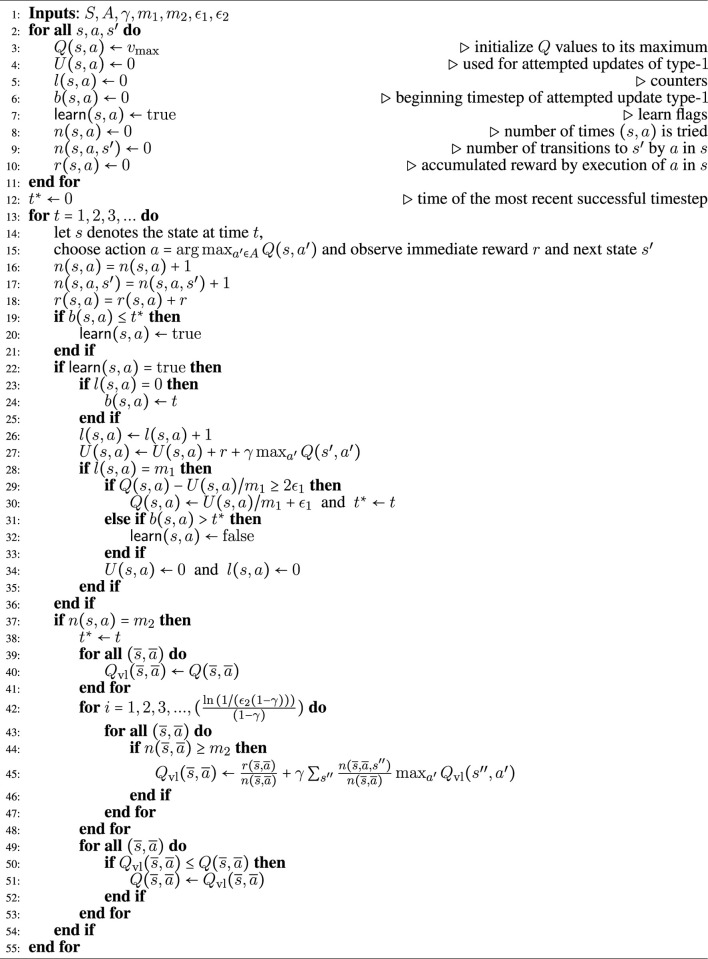

Definition 1 Consider that an RL algorithm A is executing on MDP *M*. Let *s*_*t*_ be the visited state at time step *t* and $A_{t}$ denotes the (non-stationary) policy that the A executes at *t*. For a given *ϵ* > 0 and *δ* > 0, A is a pac RL algorithm if there is an *N* > 0 such that with probability at least 1 − *δ* and for all but *N* time steps,

$$v_{M}^{A_{t}}\left(s_{t}\right)\geq v_{M}^{*}\left(s_{t}\right)-\epsilon \;$$ (2)

Eq. 2 is known as the *ϵ*-optimality condition and *N* as the *sample complexity* of A , which is a function of $\left(|S|,|A|,\frac{1}{\epsilon },\frac{1}{\delta },\frac{1}{1-\gamma }\right)$ .

Definition 2 *Consider* MDP *M* = {*S*, *A*, *R*, *T*, *γ*} *which at time* *t* *has a set of state-action value estimates* *Q*_*t*_(*s*, *a*)*, and let* *K*_*t*_ ⊆ *S* × *A* *be a set of state-action pairs labeled known. The known state-action MDP*

$$M_{K_{t}}=\left\{S\cup \left\{z_{s,a}|\left(s,a\right)\notin K_{t}\right\},A,T_{K_{t}},R_{K_{t}},\gamma \right\}$$

*is an* MDP *derived from* *M* *and* *K*_*t*_ *by defining new states* *z*_*s*,*a*_ *for each unknown state-action pair* (*s*, *a*)∉*K*_*t*_ *, with self-loops for all actions, i.e.,* $T_{K_{t}}\left(z_{s,a},\cdot ,z_{s,a}\right)=1$ *. For all* (*s*, *a*) ∈ *K*_*t*_ *, it is* $R_{K_{t}}\left(s,a\right)=R\left(s,a\right)$ *and* $T_{K_{t}}\left(s,a,\cdot \right)=T\left(s,a,\cdot \right)$ *. When an unknown state-action pair* (*s*, *a*)∉*K*_*t*_ *is experienced,* $R_{K_{t}}\left(s,a\right)=Q_{t}\left(s,a\right)\left(1-\gamma \right)$ *and the model jumps to* *z*_*s*,*a*_ *with* $T_{K_{t}}\left(s,a,z_{s,a}\right)=1$ *; subsequently,* $R_{K_{t}}\left(z_{s,a},\cdot \right)=Q_{t}\left(s,a\right)\left(1-\gamma \right)$ *.*

Let *K*_*t*_ be set of current known state-action pairs of an RL algorithm A at time *t*, and allow *K*_*t*_ to be arbitrarily defined as long as it depends only on the history of exploration data up to *t*. Any (*s*, *a*)∉*K*_*t*_ experienced at time step *t* marks an *escape event*.

Theorem 1 ((Strehl et al., 2009)). *Let* A *be a greedy* RL *algorithm for an arbitrary* MDP *M* *, and let* *K*_*t*_ *be the set of current known state-action pairs, defined based only on the history of the exploration data up to timestep* *t* *. Assume that* *K*_*t*_ = *K*_*t*+1_ *unless an update to some state-action value occurs or an escape event occurs at timestep* *t* *, and that* *Q*_*t*_(*s*, *a*) ≤ *v*_max_ *for all* (*s*, *a*) *and* *t* *. Let* $M_{K_{t}}$ *be the known state-action* MDP *at timestep* *t* *and* *π*_*t*_(*s*) = arg max_*a*_ *Q*_*t*_(*s*, *a*) *denote the greedy policy that* A *executes. Suppose now that for any positive constant* *ϵ* *and* *δ* *, the following conditions hold with probability at least* 1 − *δ* *for all* *s* *,* *a* *and* *t* *:*

***optimism:*** $v_{t}\left(s\right)\geq v_{M}^{*}\left(s\right)-\epsilon$

***accuracy:*** $v_{t}\left(s\right)-v_{M_{K_{t}}}^{\pi_{t}}\left(s\right)\leq \epsilon$

***complexity:*** *sum of number of timesteps with* *Q* *-value updates plus number of timesteps with escape events is bounded by* *ζ*(*ϵ*, *δ*) > 0*. Then, executing algorithm* A *on any* MDP *M* *will result in following a* 4*ϵ* *-optimal policy on all but*

$$O\left(\frac{\zeta \left(\epsilon ,\delta \right)}{\epsilon {\left(1-\gamma \right)}^{2}}\ln\left(\frac{1}{\delta }\right)\ln\left(\frac{1}{\epsilon \left(1-\gamma \right)}\right)\right)≃O\left(\frac{\zeta \left(\epsilon ,\delta \right)}{\epsilon {\left(1-\gamma \right)}^{2}}\right)$$ (3)

*timesteps, with probability at least* 1 − 2*δ* *.*

## 3 DDQ Algorithm

This section presents Algorithm 1, the one we call DDQ and the main contribution of this paper. Ddq integrates elements of R-max and Delayed *Q*-learning, while preserving the implementation advantages of both.

Algorithm 1 consists of four main sections: 1) In lines 1–12, the internal variables of the algorithm are initialized; 2) In lines 13–19, an action is greedily selected in the current state and the consequent immediate reward and new state are observed and recorded; 3) The model-free part of the algorithm is presented in lines 20–37 that resembles the Delayed *Q*-learning algorithm (Strehl et al., 2006); 4) Lines 38–56 represent the model-based part of the algorithm that is similar to R-max algorithm (Brafman and Tennenholtz, 2002) with a modified update mechanism which is needed for preserving the PAC property of the overall hybrid algorithm.

We refer to the assignment in line 31 of Algorithm 1 as a *type-1 update* (model-free update), and to the one on line 52 as a *type-2 update* (model-based update). The latter offers a way for new model-related information to be injected into the model-free learning process. Type-1 updates use the *m*_1_ most recent experiences (occurances) of a state-action pair (*s*, *a*) to update that pair’s value, while a type-2 update is realized through a value iteration algorithm (lines 43 − 54) and applies to state-action pairs experienced at least *m*_2_ times. The outcome at timestep *t* of the value iteration for a type-2 update is denoted $Q_{t}^{\text{vl}}\left(s,a\right)$ . The value iteration is set to run for $\frac{\ln\left(1/\left(\epsilon_{2}\left(1-\gamma \right)\right)\right)}{\left(1-\gamma \right)}$ iterations; parameter *ϵ*_2_ regulates the desired accuracy on the resulting estimate (Lemma 5). A type-1 update is successful only if the condition on line 30 of the algorithm is satisfied, and this condition ensures that the type-1 update necessarily decreases the value estimate by at least *ϵ*_1_ = 3*ϵ*_2_. Similarly, a type-2 update is successful only if the condition on line 51 of the algorithm holds. The DDQ algorithm maintains the following internal variables:

• *l* (*s*, *a*): the number of samples gathered for the update type-1 of *Q* (*s*, *a*) once *l* (*s*, *a*) = *m*_1_.
• *U* (*s*, *a*): the running sum of target values used for a type-1 update of *Q* (*s*, *a*), once enough samples have been gathered.
• *b* (*s*, *a*): the timestep at which the most recent or ongoing collection of *m*_1_ (*s*, *a*) experiences has started.
• learn(s,a) : a Boolean flag that indicates whether or not samples are being gathered for type-1 update of *Q* (*s*, *a*). The flag is set to true initially, and is reset to true whenever some Q-value is updated. It flips to false when no updates to any Q-values occurs within a time window of *m*_1_ experiences of (*s*, *a*) in which attempted updates type-1 of *Q* ^*i*^ (*s*, *a*) fail.
• *n* (*s*, *a*): variable that keeps track of the number of times (*s*, *a*) is experienced.
• *n* (*s*, *a*, *s*′): variable that keeps track of the number of transitions to *s*′ on action *a* at state *s*.
• *r* (*s*, *a*): the accumulated rewards by doing *a* in *s*.

The execution of the DDQ algorithm is tuned *via* the *m*_1_ and *m*_2_ parameters. One can practically reduce it to Delayed *Q*-learning by setting *m*_2_ very large, and to R-max by setting *m*_1_ large. The next section provides a formal proof that DDQ is not only PAC but also *possesses the minimum sample complexity* between R-max and Delayed *Q*-learning in the worst case—often, it outperforms both.

## 4 PAC Analysis of DDQ Algorithm

In general, the sample complexity of R-max and Delayed *Q*-learning is incomparable (Strehl et al., 2009); the former is better in terms of the accuracy of the resulting policy while the latter is better in terms of scaling with the size of the state space. The sample complexity of R-max algorithm is $\frac{\left|S{|}^{2}|A\right|}{\epsilon^{3}{\left(1-\gamma \right)}^{8}}$ —note the power on *ϵ*; the sample complexity of Delayed *Q*-learning algorithm is $\frac{\left|S\|A\right|}{\epsilon^{4}{\left(1-\gamma \right)}^{8}}$ —note the linear scaling with |*S*|. It appears that DDQ can bring together the best of both worlds; its sample complexity is

$$O\left(\min\left\{O\left(\frac{\left|S{|}^{2}|A\right|}{\epsilon^{3}{\left(1-\gamma \right)}^{8}}\right),O\left(\frac{\left|S\|A\right|}{\epsilon^{4}{\left(1-\gamma \right)}^{8}}\right)\right\}\right)$$

Before formally stating the PAC properties of the DDQ algorithm and proving the bound on its sample complexity, some technical groundwork needs to be laid. To slightly simplify notation, let $\kappa ≜|S\|A|\left(1+\frac{1}{\left(1-\gamma \right)\epsilon_{1}}\right)$ . Moreover, subscript *t* marks the value of a variable at the beginning of timestep *t* (particularly line 23 of the algorithm).

Definition 3 *An event when* learn(s,a)=true *and at the same time* *l*(*s*, *a*) = *m*_1_ *or* *n*(*s*, *a*) = *m*_2_ *, is called an attempted update.*

Definition 4 *At any timestep* *t* *in the execution of* DDQ *algorithm the set of known state-action pairs is defined as:*

$$K_{t}=\left\{\left(s,a\right)|n\left(s,a\right)\geq m_{2}\;or\;Q_{t}\left(s,a\right)-\left(R\left(s,a\right)+\gamma \sum_{s^{\prime }}T\left(s,a,s^{\prime }\right)v_{t}\left(s^{\prime }\right)\right)\leq 3\epsilon_{1}\right\}$$

In subsequent analysis, and to distinguish between the conditions that make a state-action pair (*s*, *a*) known, the set *K*_*t*_ will be partitioned into two subsets:

$$\begin{array}{ll} K_{t}^{1}= & \left\{\left(s,a\right)|Q_{t}\left(s,a\right)-\left(R\left(s,a\right)+\gamma \sum_{s^{\prime }}T\left(s,a,s^{\prime }\right)v_{t}\left(s^{\prime }\right)\right)\leq 3\epsilon_{1}\right\}\\ K_{t}^{2}= & \left\{\left(s,a\right)|n\left(s,a\right)\geq m_{2}\right\} \end{array}$$

Definition 5 *In the execution of* DDQ *algorithm a timestep* *t* *is called a successful timestep if at that step any state-action value is updated or the number of times that a state-action pair is visited reaches* *m*_2_ *. Moreover, considering a particular state-action pair* (*s*, *a*)*, timestep* *t* *is called a successful timestep for* (*s*, *a*) *if at* *t* *either update type-1 happens to* *Q*(*s*, *a*) *or the number of times that* (*s*, *a*) *is visited reaches* *m*_2_ *.*

Recall that a type-1 update necessarily decreases the Q-value by at least *ϵ*_1_. Defining rewards as positive quantities prevents the Q-values from becoming negative. At the same time, state-action pairs can initiate update type-2 only once they are experienced *m*_2_ times. Together, these conditions facilitate the establishment of an upper-bound on the total number of successful timesteps during the execution of DDQ:

Lemma 1 *The number of successful timesteps for a particular state-action pair* (*s*, *a*) *in a* DDQ *algorithm is at most* $1+\frac{1}{\left(1-\gamma \right)\epsilon_{1}}$ *. Moreover, the total number of successful timesteps is bounded by* *κ* *.*

*Proof*. See Supplementary Appendix S1.

Lemma 2 *The total number of attempted updates in* DDQ *algorithm is bounded by* |*S*‖*A*|(1 + *κ*)*.*

*Proof*. See Supplementary Appendix S2.

Lemma 3 *Let* *M* *be an* MDP *with a set of known state-action pairs* *K*_*t*_ *. If we assume that for all state-action pairs* (*s*, *a*)∉*K*_*t*_ *we have* $Q_{t}\left(s,a\right)\leq \frac{1}{1-\gamma }$ *, then for all state-action pairs in the known state-action* MDP $M_{K_{t}}$ *it holds*

$$Q_{M_{K_{t}}}^{*}\left(s,a\right)\leq \frac{1}{1-\gamma }$$

*Proof*. See Supplementary Appendix S3.

Choosing *m*_1_ big enough and applying Hoefding’s inequality allows the following conclusion (Lemma 4) for all type-1 updates, and paves the way for establishing the optimism condition of Theorem 1.

Lemma 4 *Suppose that at time* *t* *during the execution of* DDQ *a state-action pair* (*s*, *a*) *experiences a successful update of type-1 with its value changing from* *Q*(*s*, *a*) *to* *Q*′(*s*, *a*)*, and that there exists* $\exists \epsilon_{2}\in \left(0,\frac{\epsilon_{1}}{2}\right)$ *such that* *∀s* ∈ *S* *and* *∀t*′ < *t* *,* $v_{t^{\prime }}\left(s\right)\geq v_{M}^{*}\left(s\right)-2\epsilon_{2}$ *. If*

$$m_{1}\geq \frac{\ln\left(\frac{8|S\|A|\left(1+\kappa \right)}{\delta }\right)}{2{\left(\epsilon_{1}-2\epsilon_{2}\right)}^{2}{\left(1-\gamma \right)}^{2}}≃O\left(\frac{\ln\left(\frac{|S{|}^{2}|A{|}^{2}}{\delta }\right)}{\epsilon_{1}^{2}{\left(1-\gamma \right)}^{2}}\right)$$ (4)

*for* *κ* = |*S*‖*A*|(1 + 1/(1 − *γ*)*ϵ*_1_)*, then* $Q^{\prime }\left(s,a\right)\geq Q_{M}^{*}\left(s,a\right)$ *with probability at least* $1-\frac{\delta }{8}$ *.*

*Proof*. In Supplementary Appendix S4.

The following two lemmas are borrowed from (Strehl et al., 2009) with very minor modifications, and inform on how to choose parameter *m*_2_, and the number of iterations for the value iteration part of the DDQ algorithm in order to obtain a desired accuracy.

Lemma 5 (cf. (Strehl et al., 2009, *Proposition 4)*) *Suppose the value-iteration algorithm runs on* MDP *M* *for* $\frac{\ln\left(1/\epsilon_{2}\left(1-\gamma \right)\right)}{1-\gamma }$ *iterations, and each state-action value estimate* *Q*(*s*, *a*) *is initialized to some value between 0 and* *v*_max_ *for all states and actions. Let* *Q*′(*s*, *a*) *be the state-action value estimate the algorithm yields. Then* $\max_{s,a}\left\{\left|Q^{\prime }\left(s,a\right)-Q_{M}^{*}\left(s,a\right)\right|\right\}\leq \epsilon_{2}$ *.*

Lemma 6 *Consider an* MDP *M* *with reward function* *R* *and transition probabilities* *T* *. Suppose another* MDP $\hat{M}$ *has the same state and action set as* *M* *, but maintains a maximum likelihood* (ml) *estimate of* *R* *and* *T* *, with* *n*(*s*, *a*) ≥ *m*_2_ *, in the form of* $\hat{R}$ *and* $\hat{T}$ *respectively. With* *C* *a constant and for all state-action pairs, choosing*

$$m_{2}\geq C\left(\frac{|S|+\ln\left(8|S\|A|/\delta \right)}{\epsilon_{2}^{2}{\left(1-\gamma \right)}^{4}}\right)≃O\left(\frac{|S|+\ln\left(|S\|A|/\delta \right)}{\epsilon_{2}^{2}{\left(1-\gamma \right)}^{4}}\right)$$

guarantees

$$\begin{array}{ll} \left|R\left(s,a\right)-\hat{R}\left(s,a\right)\right| & \leq C\epsilon_{2}{\left(1-\gamma \right)}^{2}\\ \|T\left(s,a,\cdot \right)-\hat{T}\left(s,a,\cdot \right){\|}_{1} & \leq C\epsilon_{2}{\left(1-\gamma \right)}^{2} \end{array}$$

*with probability at least* $1-\frac{\delta }{8}$ *. Moreover, for any policy* *π* *and for all state-action pairs,*

$$\begin{array}{ll} \left|Q_{M}^{\pi }\left(s,a\right)-Q_{\hat{M}}^{\pi }\left(s,a\right)\right| & \leq \epsilon_{2}\\ \left|v_{M}^{\pi }\left(s\right)-v_{\hat{M}}^{\pi }\left(s\right)\right| & \leq \epsilon_{2} \end{array}$$

*with probability at least* $1-\frac{\delta }{8}$ *.*

*Proof*. Combine (Strehl et al., 2009, Lemmas 12–15).

Lemma 7 *Let* *t*_1_ < *t*_2_ *be two timesteps during the execution of the* DDQ *algorithm. If*

$$Q_{t_{1}}\left(s,a\right)\geq Q_{M_{K_{t_{1}}^{2}}}^{*}\left(s,a\right)-2\epsilon_{2}\;\forall \left(s,a\right)\in S\times A$$

*then with probability at least* $1-\frac{\delta }{8}$

$$Q_{M_{K_{t_{1}}^{2}}}^{*}\left(s,a\right)\geq Q_{M_{K_{t_{2}}^{2}}}^{*}\left(s,a\right)\;\forall \left(s,a\right)\in S\times A$$

*Proof*. See Supplementary Appendix S5.

Lemma 5 and Lemma 6 together have as a consequence the following Lemma, which contributes to establishing the accuracy condition of Theorem 1 for the DDQ algorithm.

Lemma 8 *During the execution of* DDQ *, for all* *t* *and* (*s*, *a*) ∈ *S* × *A* *, we have:*

$$Q_{M_{K_{t}^{2}}}^{*}\left(s,a\right)-2\epsilon_{2}\leq Q_{t}\left(s,a\right)\leq Q_{M_{K_{t}^{2}}}^{*}\left(s,a\right)+2\epsilon_{2}$$ (5)

*with probability at least* $1-\frac{3\delta }{8}$ *.*

*Proof*. See Supplementary Appendix S6.

Lemma 1 has already offered a bound on the number of updates in DDQ; however, for the complexity condition of Theorem 1 to be satisfied, one needs to show that during the execution of Algorithm 1 the number of escape events is also bounded. The following Lemma is the first step: it states that by picking *m*_1_ as in (4), and under specific conditions, an escape event necessarily results in a successful type-1 update. With the number of updates bounded, Lemma 9 can be utilized to derive a bound on the number of escape events.

Lemma 9 *With the choice of* *m*_1_ *as in* (4) *, and assuming the* DDQ *algorithm at timestep* *t* *with* (*s*, *a*)∉*K*_*t*_ *,* *l*(*s*, *a*) = 0 *and* learn(s,a)=true *, we know that an attempted type-1 update of* *Q*(*s*, *a*) *will necessarily occur within* *m*_1_ *occurrences of* (*s*, *a*) *after* *t* *, say at timestep* $t_{m_{1}}$ *. If* (*s*, *a*) *has been visited fewer than* *m*_2_ *till* $t_{m_{1}}$ *, then the attempted type-1 update at* $t_{m_{1}}$ *will be successful with probability at least* $1-\frac{\delta }{8}$ *.*

*Proof*. See Supplementary Appendix S7.

Lemma 10 *Let* *t* be *the timestep when* (*s*, *a*) *has been visited for* *m*_1_ *times after the conditions of* Lemma 9 *were satisfied. If the update at timestep* *t* *is unsuccessful and at timestep* *t* + 1 *it is* learn(s,a)=false *, then* (*s*, *a*) ∈ *K*_*t*+1_ *.*

*Proof*. See Supplementary Appendix S8.

A bound on the number the escape events of DDQ algorithm can be derived in a straightforward way. Note that a state-action pair that is visited *m*_2_ times becomes a permanent member of set *K*_*t*_. Therefore, the number of escape events is bounded by |*S*‖*A*|*m*_2_. On the other hand, Lemma 9 and the learn flag mechanism (i.e. Lemma 10) suggest another upper bound on escape events. The following Lemma simply states an upper bound for escape events in DDQ as the minimum among the two bounds.

Lemma 11 *During the execution of* DDQ *, with the assumption that* Lemma 9 *holds, the total number of timesteps with* (*s*_*t*_, *a*_*t*_)∉*K*_*t*_ (*i.e. escape events*) *is at most* $\min\left\{\left.2m_{1}\kappa ,|S\|A|m_{2}\right)\right\}$ *.*

*Proof*. See Supplementary Appendix S9.

Next comes the main result of this paper. The statement that follows establishes the PAC properties of the DDQ algorithm and provides a bound on its sample complexity.

Theorem 2 *Consider an* MDP *M* = {*S*, *A*, *T*, *R*, *γ*}*, and let* $\epsilon \in \left(0,\frac{1}{1-\gamma }\right)$ *, and* *δ* ∈ (0, 1)*. There exist* $m_{1}=O\left(\ln\left(|S{|}^{2}|A{|}^{2}/\delta \right)/\epsilon_{1}^{2}{\left(1-\gamma \right)}^{2}\right)$ *and* $m_{2}=O\left(|S|+\ln\left(|S\|A|/\delta \right)/\epsilon_{2}^{2}{\left(1-\gamma \right)}^{4}\right)$ *with* $\frac{1}{\epsilon_{1}}=\frac{3}{\left(1-\gamma \right)\epsilon }=O\left(1/\epsilon \left(1-\gamma \right)\right)$ *and* $\epsilon_{2}=\frac{\epsilon_{1}}{3}$ *, such that if* DDQ *algorithm is executed,* *M* *follows a* 4*ϵ* *-optimal policy with probability at least* 1 − 2*δ* *on all but*

$$O\left(\min\left\{O\left(|S{|}^{2}|A|/\epsilon^{3}{\left(1-\gamma \right)}^{8}\right),O\left(|S\|A|/\epsilon^{4}{\left(1-\gamma \right)}^{8}\right)\right\}\right)$$

timesteps (logarithmic factors ignored).

Proof. We intend to apply Theorem 1. To satisfy the *optimism* condition, we start by proving that $Q_{t}\left(s,a\right)\geq Q_{M}^{*}\left(s,a\right)-2\epsilon_{2}$ by strong induction for all state-action pairs:

1) At *t* = 1, the value of all state-action pairs are set to the maximum possible value in MDP *M*. This implies that $Q_{1}\left(s,a\right)\geq Q_{M}^{*}\left(s,a\right)\geq Q_{M}^{*}\left(s,a\right)-2\epsilon_{2}$ , therefore $v_{t}\left(s\right)\geq v_{M}^{*}\left(s\right)-2\epsilon_{2}$ . 2) Assume that $Q_{t}\left(s,a\right)\geq Q_{M}^{*}\left(s,a\right)-2\epsilon_{2}$ holds for all timesteps before or equal to *t* = *n* − 1. 3) At timestep *t* = *n*, all $\left(s,a\right)\notin K_{n}^{2}$ can only be updated by a type-1 update before or at *t* = *n*. For these state-action pairs, Lemma 4 implies that it will be $Q_{n}\left(s,a\right)\geq Q_{M}^{*}\left(s,a\right)$ with probability $1-\frac{\delta }{8}$ .

For all $\left(s,a\right)\in K_{n}^{2}$ , on the other hand, by Lemma 8 and with probability $1-\frac{3\delta }{8}$ :

$$Q_{n}\left(s,a\right)\geq Q_{M_{K_{n}^{2}}}^{*}\left(s,a\right)-2\epsilon_{2}\geq Q_{M}^{*}\left(s,a\right)-2\epsilon_{2}$$

Note that $Q_{M_{K_{n}^{2}}}^{*}\left(s,a\right)\geq Q_{M}^{*}\left(s,a\right)$ since $M_{K_{n}^{2}}$ is similar to *M* exept for $\left(s,a\right)\notin K_{n}^{2}$ which their values are set to be more than or equal to $Q_{M}^{*}\left(s,a\right)$ . Therefore, $Q_{t}\left(s,a\right)\geq Q_{M}^{*}\left(s,a\right)-2\epsilon_{2}$ holds for all timesteps *t* and all state-action pairs, which directly implies $v_{t}\left(s\right)\geq v_{M}^{*}\left(s\right)-2\epsilon_{2}\geq v_{M}^{*}\left(s\right)-\epsilon$ .

To establish the *accuracy* condition, first write

$$Q_{t}\left(s,a\right)=R\left(s,a\right)+\gamma \underset{s^{\prime }}{\sum }T\left(s,a,s^{\prime }\right)\underset{a^{\prime }}{\max}Q_{t}\left(s^{\prime },a^{\prime }\right)+\beta \left(s,a\right)$$ (6)

If (*s*, *a*) ∈ *K*_*t*_, there can be two cases: either $\left(s,a\right)\in K_{t}^{1}$ or $\left(s,a\right)\in K_{t}^{2}$ . If $\left(s,a\right)\in K_{t}^{1}$ , then by Definition 4 *β*(*s*, *a*) ≤ 3*ϵ*_1_. If $\left(s,a\right)\in K_{t}^{2}$ , then Lemma 8 (right-hand side inequality) implies that with probability at least $1-\frac{3\delta }{8}$

$$2\epsilon_{2}\geq Q_{t}\left(s,a\right)-Q_{M_{K_{t}^{2}}}^{*}\left(s,a\right)$$ (7)

Meanwhile,

$$Q_{M_{K_{t}^{2}}}^{*}\left(s,a\right)=R\left(s,a\right)+\gamma \underset{s^{\prime }}{\sum }T\left(s,a,s^{\prime }\right)\underset{a^{\prime }}{\max}Q_{M_{K_{t}^{2}}}^{*}\left(s^{\prime },a^{\prime }\right)$$ (8)

and substituting from (8) and (6) into (7) yields

$$\gamma \underset{s^{\prime }}{\sum }T\left(s,a,s^{\prime }\right)\left(\underset{a^{\prime }}{\max}Q_{t}\left(s^{\prime },a^{\prime }\right)-\underset{a^{\prime }}{\max}Q_{M_{K_{t}^{2}}}^{*}\left(s^{\prime },a^{\prime }\right)\right)+\beta \left(s,a\right)\leq 2\epsilon_{2}$$ (9)

Let $a_{1}≔\arg\max_{a^{\prime }}Q_{M_{K_{t}^{2}}}\left(s^{\prime },a^{\prime }\right)$ and bound the difference

$$\begin{array}{ll} \underset{a^{\prime }}{\max}Q_{t}\left(s^{\prime },a^{\prime }\right)-\underset{a^{\prime }}{\max}Q_{M_{K_{t}^{2}}}^{*}\left(s^{\prime },a^{\prime }\right) & =\underset{a^{\prime }}{\max}Q_{t}\left(s^{\prime },a^{\prime }\right)-Q_{M_{K_{t}^{2}}}^{*}\left(s^{\prime },a_{1}\right)\\ & \geq Q_{t}\left(s^{\prime },a_{1}\right)-Q_{M_{K_{t}^{2}}}^{*}\left(s^{\prime },a_{1}\right) \end{array}$$

Apply Lemma 8 (left-hand side inequality) to the latter expression to get

$$\underset{a^{\prime }}{\max}Q_{t}\left(s^{\prime },a^{\prime }\right)-\underset{a^{\prime }}{\max}Q_{M_{K_{t}^{2}}}^{*}\left(s^{\prime },a^{\prime }\right)\geq -2\epsilon_{2}$$

which implies for (9) that

$$2\epsilon_{2}\geq \beta \left(s,a\right)-2\gamma \epsilon_{2}\;\Rightarrow \;\beta \left(s,a\right)\leq 2\left(1+\gamma \right)\epsilon_{2}\leq 3\epsilon_{2}$$

Thus in any case when (*s*, *a*) ∈ *K*_*t*_, *β*(*s*, *a*) ≤ 3*ϵ*_1_ with probability at least $1-\frac{3\delta }{8}$ . In light of this, considering a policy dictating actions *a* = *π*_*t*_(*s*) and mirroring (6)–(8) we write for the values of states in which $\left(s,\pi_{t}\left(s\right)\right)\in K_{t}$

$$\begin{array}{ll} v_{M_{K_{t}}}^{\pi_{t}}\left(s\right) & =R\left(s,\pi_{t}\left(s\right)\right)+\gamma \underset{s^{\prime }}{\sum }T\left(s,\pi_{t}\left(s\right),s^{\prime }\right)v_{M_{K_{t}}}^{\pi_{t}}\left(s^{\prime }\right)\\ v_{t}\left(s\right) & =R\left(s,\pi_{t}\left(s\right)\right)+\gamma \underset{s^{\prime }}{\sum }T\left(s,\pi_{t}\left(s\right),s^{\prime }\right)v_{t}\left(s^{\prime }\right)+\beta \left(s,a\right) \end{array}$$

while for those in which $\left(s,\pi_{t}\left(s\right)\right)\notin K_{t}$ , we already know that

$$\begin{array}{ll} v_{M_{K_{t}}}^{\pi_{t}}\left(s\right) & =Q_{t}\left(s,\pi_{t}\left(s\right)\right)\\ v_{t}\left(s\right) & =Q_{t}\left(s,\pi_{t}\left(s\right)\right) \end{array}$$

So now if one denotes

$$\alpha ≔\underset{s}{\max}\left(v_{t}\left(s\right)-v_{M_{K_{t}}}^{\pi_{t}}\left(s\right)\right)=v_{t}\left(s^{*}\right)-v_{M_{K_{t}}}^{\pi_{t}}\left(s^{*}\right)$$

then either *α* = 0 (when $\left(s,\pi_{t}\left(s\right)\right)\notin K_{t}$ ) or it affords an upper bound

$$\begin{array}{c} \gamma \underset{s^{\prime }}{\sum }T\left(s^{*},\pi_{t}\left(s^{*}\right),s^{\prime }\right)\left(v_{t}\left(s^{\prime }\right)-v_{M_{K_{t}}}^{\pi_{t}}\left(s^{\prime }\right)\right)+\beta \left(s^{*},\pi_{t}\left(s^{*}\right)\right)\\ \leq \gamma \underset{s^{\prime }}{\sum }T\left(s^{*},\pi_{t}\left(s^{*}\right),s^{\prime }\right)\left(v_{t}\left(s^{\prime }\right)-v_{M_{K_{t}}}^{\pi_{t}}\left(s^{\prime }\right)\right)+3\epsilon_{1}\leq \gamma \alpha +3\epsilon_{1} \end{array}$$

from which it follows that $\alpha \leq \gamma \alpha +3\epsilon_{1}\;\Rightarrow \;\alpha \leq \frac{3\epsilon }{1-\gamma }=\epsilon$ .

Finally, to analyze *complexity* invoke Lemma 1 and Lemma 11 to see that the learning complexity *ζ*(*ϵ*, *δ*) is bounded by *κ* + min (2*m*_1_ *κ*, |*S*‖*A*|*m*_2_) with probability $1-\frac{\delta }{8}$ .

In conclusion, the conditions of Theorem 2 are satisfied with probability 1 − *δ* and therefore the DDQ algorithm is PAC. Substituting *ζ*(*ϵ*, *δ*) into (3) completes the proof.

## 5 Numerical Results

This section opens with a comparison of the DDQ algorithm to its parent technologies. It proceeds with additional comparisons to the state-of-the-art in both model-based (Szita and Szepesvá ri, 2010) as well as model-free (Dong et al., 2019) RL algorithms. For this comparison, the algorithms with the currently best sample complexity are implemented on a type of MDP which has been proposed and used in literature as a model which is objectively difficult to learn (Strehl et al., 2009). Experimental implementation and performance evaluation for DDQ deployed in the context of the motivating pediatric rehabilitation application is also presented, illustrating the possible advantages of DDQ over direct human control in real-world applications.

### 5.1 Comparison of DDQ With Its Parent Methodologies

**FIGURE 1:**
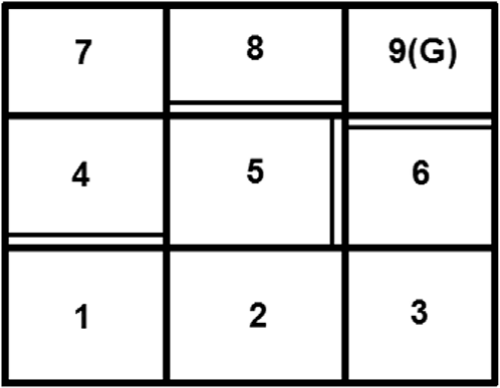
The grid-world example.

**FIGURE 2:**
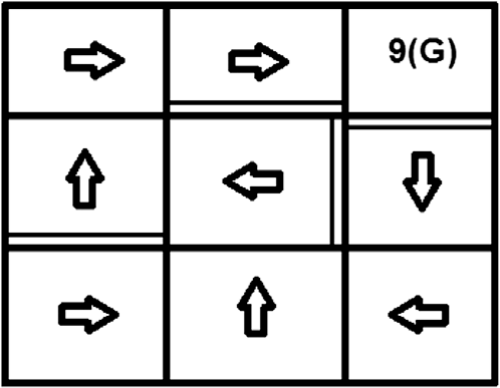
The actual optimal policy in the grid-world example.

The first round of comparisons start with R-max, Delayed Q-learning, and DDQ being implemented on a small-scale grid-world example (Figure 1). This example test case has nine states, with the initial state being the one labeled 1, and the terminal (goal) state labeled 9. Each state is assigned a reward of 0 except for the terminal state which has 1. For this example, *γ*: = 0.8. In all states but the terminal one, the system has four primitive actions available: down (d) , left (l) , up (u) , and right (r) . The grid-world of Figure 1 includes cells with two types of boundaries: the boundaries marked with a single-line afford transition probabilities of 0.9 through them; the boundaries marked with a double line afford transitions through them at probability 0.1. The optimal policy for this grid-world example is shown in Figure 2.

Initializing the three PAC algorithms with parameters *m*_1_ = 65, *m*_2_ = 175 and *ɛ* = 0.06, yields the performance metrics shown in Table 1, which are measured in terms of the number of samples needed to reach at 4*ɛ* optimality, averaged over 10 algorithm runs. Parameters *m*_1_ and *m*_2_ are intentionally chosen to enable a fair comparison, in the sense that the sample complexity of the model-free Delayed Q-learning, and the model-based R-max algorithms are almost identical. In this case, and with these same tuning parameters, DDQ yields a modest but notable sample complexity improvement.

**TABLE 1:** Average # of samples for reaching 4*ɛ* optimality.

Algorithms	# Of samples
Delayed Q-learning	6622
R-max	6727
ddq	5960

### 5.2 Comparison of DDQ to the Best Known PAC RL Algorithms

The lowest known bound on the sample complexity of a model-based RL algorithm on a infinite-horizon MDP is |*S*‖*A*|/*ϵ* ^2^ (1 − *γ*)^6^ (by the Mormax algorithm (Szita and Szepesvári, 2010)). For the model-free case (again on a infinite-horizon MDP), the lowest bound on the sample complexity is |*S*‖*A*|/*ϵ* ^2^ (1 − *γ*)^7^, achieved by UCB Q-learning (Dong et al., 2019) (the extended version of (Jin et al., 2018) which is for finite-horizon MDP).

**FIGURE 3:**
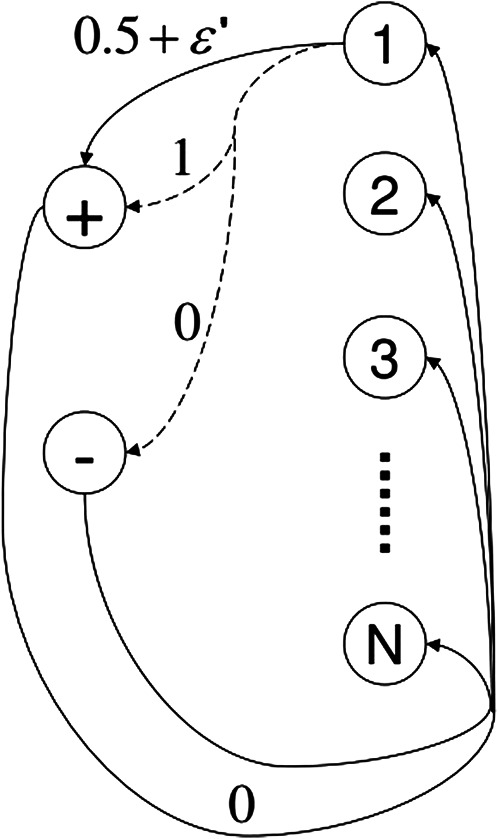
A family of difficult-to-learn mdps (Strehl et al., 2009).

To perform a fair and meaningful comparison of these algorithms to DDQ, consider a family of “difficult-to-learn” MDP as Figure 3. The MDP has *N* + 2 states as *S* = {1, 2, …, *N*, +, − }, and *A* different actions. Transitions from each state *i* ∈ {1, …, *N*} are the same, so only transitions from state 1 are shown. One of the actions (marked by solid line) deterministically transports the agent to state + with reward 0.5 + *ϵ*′ (with *ϵ*′ > 0). Let *a* be any of the other *A*− 1 actions (represented by dashed lines). From any state *i* ∈ {1, …, *N*}, taking action *a* will trigger a transition to state + with reward 1 and probability *p*_*ia*_, or to state − with reward 0 and probability 1 − *p*_*ia*_, where *p*_*ia*_ ∈ {0.5, 0.5 + 2*ϵ*′} are numbers very close to 0.5 + *ϵ*′. For each state *i* ∈ {1, …, *N*}, there is at most one *a* such that *p*_*ia*_ = 0.5 + 2*ϵ*′. Transitions from states + and − are identical; they simply reset the agent to one of the states {1, …, *N*} uniformly at random.

For an MDP such as the one shown in Figure 3, the optimal action in any state *i* ∈ {1, …, *N*} is independent of the other states; specifically, it is the action marked by the solid arrow if *p*_*ia*_ = 0.5 for all dashed actions *a*, or the action marked by the dashed arrow for which *p*_*ia*_ = 0.5 + 2*ϵ*′, otherwise. Intuitively, this MDP is hard to learn for exactly the same reason that a biased coin is hard to be recongized as such if its bias (say, the probability of landing on heads) is close to 0.5 (Strehl et al., 2009).

We thus try to learn such an MDP *M* with *N* = 2, *A* = 2, and *ϵ*′ = 0.04. The accuracy that the learned policy should satisfy is set to *ϵ* = 0.002 5, and the probability of failure is set to *δ* = 0.01. Results are averaged over 50 runs of each algorithm running on MDP *M*.

**FIGURE 4:**
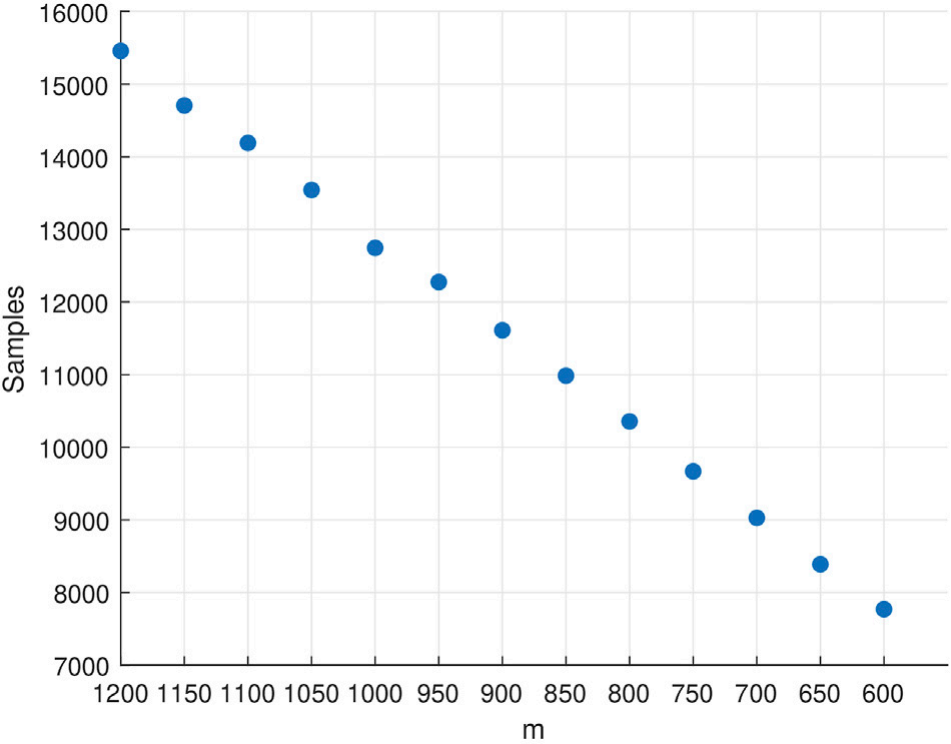
The number of samples required by the Mormax algorithm.

We empirically fine-tune the parameters of Mormax and UCB Q-learning algorithms to maximize their performance on learning the near optimal (4*ϵ*-optimal) policy of *M* in terms of the required samples. As expected, the required samples decrease (almost linearly) in *m* (Figure 4) until the necessary condition for the convergence of the algorithm is violated (at around *m* = 600). For that reason, we cap *m* at 600 which requires 7,770 samples on average for Mormax to learn the optimal policy. Yet another important performance metric to record for a model-based RL algorithm is the number of times it needs resolve the learned model through value-iteration, since the associated computational effort is highly dependent on this number. For Mormax, the average number of times it performs model resolution is 12.06.

**FIGURE 5:**
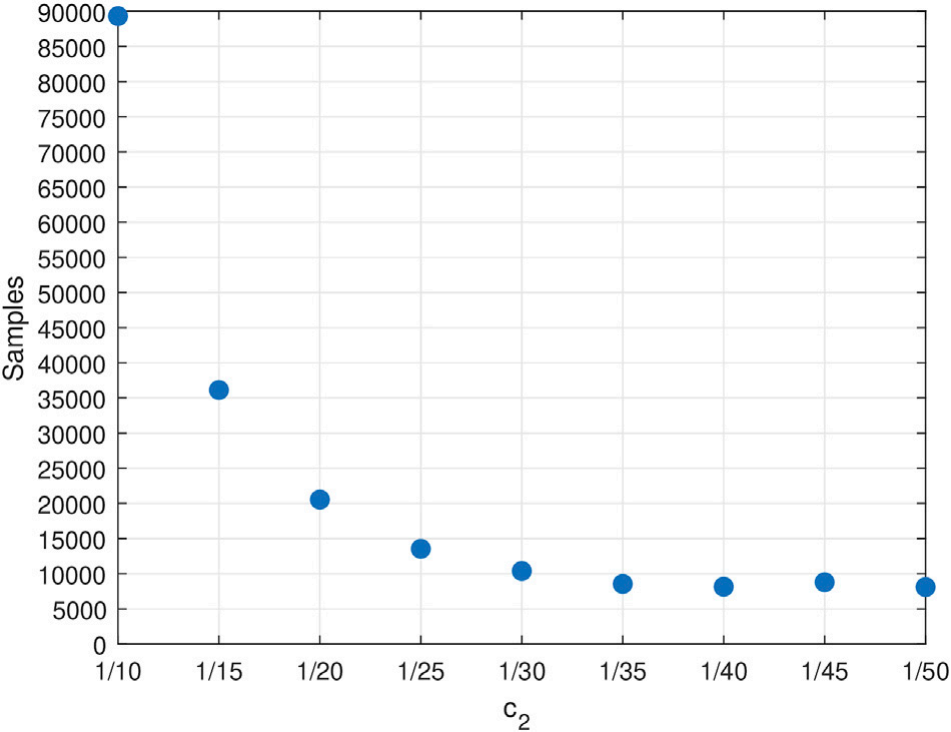
The required samples by UCB Q-learning algorithm.

The performance of the UCB Q-learning algorithm appears to be very sensitive to its *c*_2_ parameter. The value of $4\sqrt{2}$ that has been suggested for *c*_2_ (Dong et al., 2019) proved very conservative, with the algorithm sometimes requiring millions of data for converging to the optimal policy on *M*. The reason is that values of *c*_2_ that high cause the effective updates to start when the learning rate has already become very small, thus slowing down the convergence speed. We therefore tune the UCB Q-learning algorithm to achieve maximum performance on *M* by setting its parameter *c*_2_ = 1/50 (see Figure 5); with this setting, the algorithm requires 8,097 samples to learn the optimal policy on average. Setting *c*_2_ < 1/50 may cause the algorithm to lie outside the upper confidence interval, and as a result, the algorithm either requires an actual higher number of samples or it fails to convege altogether to the optimal policy after 10^6^ samples.

We compare the best performance we could achieve with Mormax and UCB Q-learning with that of DDQ which we tune with *m*_1_ = 150 and *m*_2_ = 750. The average required samples required by DDQ for learning the 4*ϵ*-optimal policy on *M* is 5662, while the number of times that the R-max component of the algorithm resolves the model through value-iteration part is 3.76 on average.

Thus, although the provable worst-case bound on the sample complexity of DDQ algorithm appears higher than that of Mormax and UCB Q-learning (cf. (Jaksch et al., 2010) for a slightly worse bound), DDQ can outperform both algorithms in terms of the required data samples, especially in difficult learning tasks. What is more, the hybrid nature of DDQ algorithm enables significant savings in terms of computational effort—the latter captured by the number of times when the algorithm resorts to model resolution—compared to model-based algorithms like Mormax. Table 2 summarizes the results of this comparison.

**TABLE 2:** The best possible performance on learning mdp *M*.

Algorithms	# Of samples	# Of model resolution
Mormax	7770	12.06
UCB Q-learning	8097	0 (model-free)
ddq	5662	3.76

### 5.3 Experimental Results

Early development in humans is highly dependent to the ability of infants to explore their surrounding physical environment and use the exploration experiences to learn (Campos et al., 2000; Clearfield, 2004; Walle and Campos, 2014; Adolph, 2015). With this given, children with motor delay and disability (such as, for example those diagnosed with Down syndrome (Palisano et al., 2001; Cardoso et al., 2015)) have significantly fewer opportunities for self-initiated environment exploration, but also social interactions with their peers which are also expected to occur and develop within this environment. This is presumably why a portion of the research on pediatric rehabilitation has considered HRI as a way to partially compensate for the dearth of social interaction and a means for improvement of social skills in infants who face communication challenges (Feil-Seifer and Mataric, 2009; Scassellati et al., 2012; Sartorato et al., 2017). These studies suggest, for example, that children with autism may socially engage in play activities with interactive robots, and even sometimes prefer this type of interaction over that with adults or computer games (Kim et al., 2013). Within the pediatric rehabilitation paradigm, HRI scenarios are designed by considering infants’ abilities and interests based on their age and level of impairment (Prosser et al., 2012; Pereira et al., 2013; Adolph, 2015). While many interesting aspects of the HRI problem in the context of pediatric rehabilitation can be considered, one driving objective behind the work presented in this paper is to design *automated decision-making* algorithms for robots when they socially interact with children, in order to keep them interested and engaged in the type of activities and behavior that are considered beneficial for the purposes of rehabilitation.

As mentioned in Section 1, the motivating application behind the particular approach described in this paper is that of (early) pediatric motor rehabilitation that leverages social child-robot interaction within play-based activities. In principle, the objective of these targeted activities is to encourage and sustain goal-driven physical activity, i.e., mobility, on the part of the child, with the understanding that such mobility will help the infant explore not only her environment, but also the latent capabilities of her own body. In this area, robot automation can serve by reducing the stress, cognitive load, and dedicated time requirements of human caregivers by allowing the robots to become more independent children playmates. To gain autonomy, a purposeful robot playmate needs an automated decision-making algorithm that will allow it to learn what to do to sustain and extend playtime. This is particularly challenging for a whole range of reasons. First, this is not a “one-size-fits-all” solution—every human playmate is behaviorally different from another, necessitating an ability on the part of the robot to adapt and personalize its own behavior and response to the child it is interacting with. In addition, especially when it comes to algorithms learning from data and particularly because every human subject is fundamentally different in terms of social interaction preferences, the data pool will invariably be very small and sparse (Zehfroosh et al., 2017; Kokkoni et al., 2020). There will always be little prior information about the infant’s preferences, and the usually limited time of infant’s rehabilitation sessions hardly provides sufficient data for machine learning algorithms. Methods that are able to better handle sparsity in training data are therefore expected to perform better than alternatives.

In terms of the mathematical model of HRI, partially observable Markov decision process (POMDP) is the most common Markovian model because some internal parameters of the human partners such as intent are not directly observable (Broz et al., 2013; Ognibene and Demiris, 2013; Mavridis, 2015). Dealing with POMDP is computationally demanding and it usually requires large amounts of data for learning (Bernstein et al., 2002). This is the reason that whenever a particular HRI application allows for some legitimate simplifying assumptions, researchers have tried to stick to less complex Markovian models such as a mixed observability Markov decision process (MOMDP) (Bandyopadhyay et al., 2013; Nikolaidis et al., 2014) or an MDP (Keizer et al., 2013; McGhan et al., 2015). For the motivating application of this paper (i.e. pediatric rehabilitation) an MDP appears to be a more appropriate choice since it possesses fewer parameters and hence presumably requires smaller bodies of data in order to train (Zehfroosh et al., 2017).

In terms of the learning algorithm itself, it needs to be particularly efficient in its data utilization, and preferably be able to guarantee some level of performance even when the training dataset is small. The presented hybrid RL algorithm DDQ seems a good fit for the application described above as its hybrid structure promotes data efficiency and its performance is also backed up with theoretical guarantee.

**FIGURE 6:**
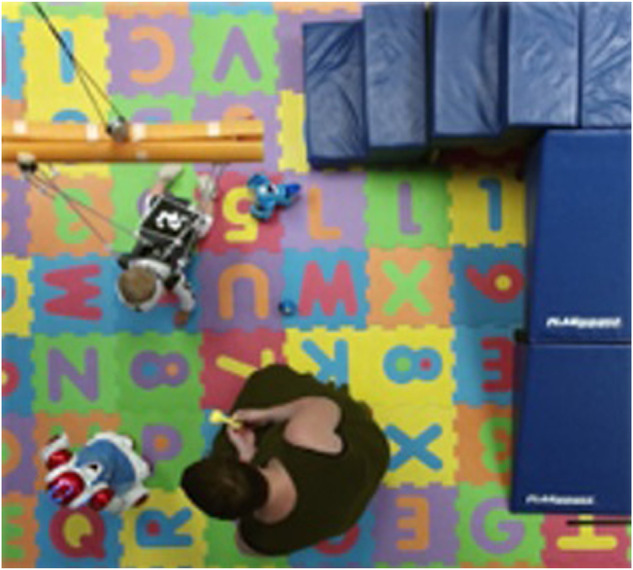
Instance of play-based child-robot social interaction. Two robots are visible in the scene: a small humanoid nao, and a differential-drive small mobile robot toy Dash.

This section presents some outcomes related to the performance of DDQ in a pediatric rehabilitation session like the one described above. Figure 6 shows a robot-assisted motor rehabilitation environment for infants involving two robots (NAO and Dash) engaged in free-play activities with an infant.

**FIGURE 7:**
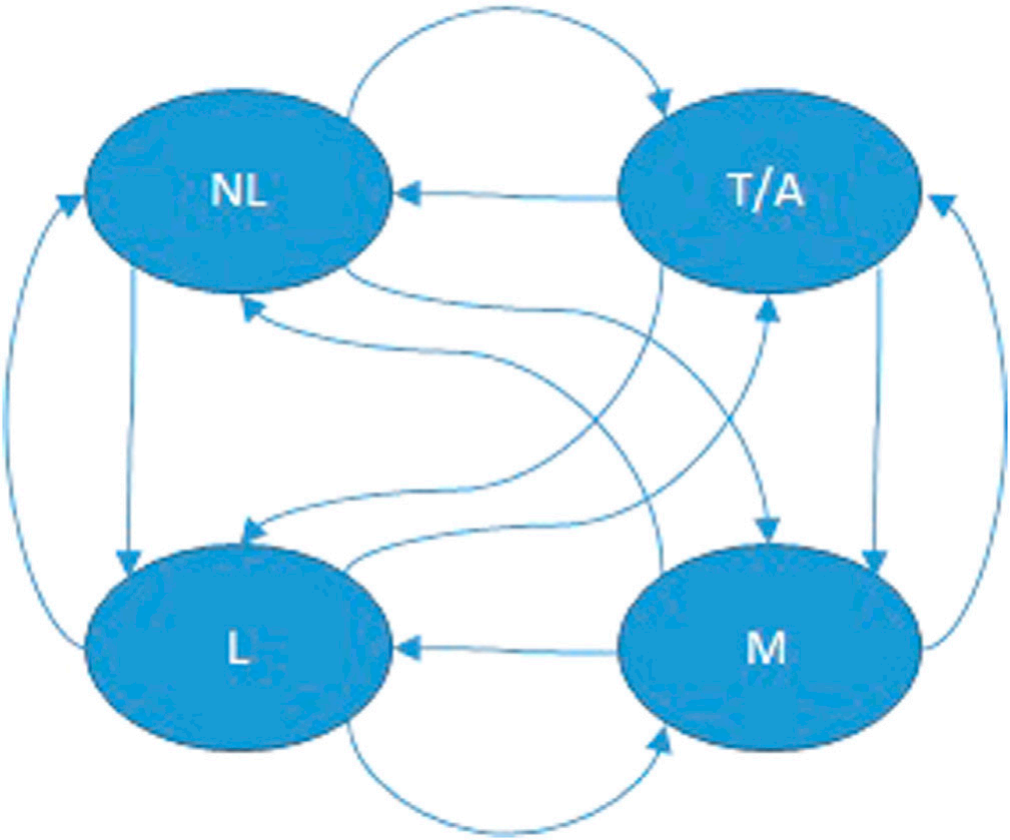
MDP model for the game of chase between a mobile robot and an infant.

The proposed MDP model for the case of a simple chasing game is shown in Figure 7. In this MDP, the state set is S={NL,L,T/A,M} , where NL represents the state where the child is not looking at the robot, L is expressed with the state when the infant is looking at the robot but not chasing it, T/A denotes circumstances when the child is touching the robot or showing some form of excitement (e.g., clapping, laughing, squealing etc), and M stands for the situation when the child is chasing the robot. The action set for the robot is A={cd,s/tu,id} . Here, cd stands for the robot closing its distance to the infant, s/tu corresponds to the robot preserving its distance to the infant while, say, standing still or rotating around her, and id represents the case where the robot is increasing its distance to the child. Transitions in the graph of Figure 7 can be labeled by one of the aforementioned actions, and annotated with the transition probabilities associated with each action (Note that in practice these robot actions generally have nondeterministic outcomes.) In the described MDP model, transitions are expressing the infant’s reactions to the robot’s action. With respect to the overarching rehabilitation objectives for the social interaction between infant and robot, the favorable states to reach in this game are T/A and M . These states are assigned a high (er) reward of 0.5 and 1, respectively. The reward for all other states is set to 0.

The chasing game is played with Dash as (a small) part of six 1-h infant-robot social interaction sessions with a 10 month old subject, and data in the form of video are collected and annotated. In the six sessions the robot was remotely controlled and its actions were chosen by a human operator who was observing the interaction. The DDQ algorithm was trained on the data from these six sessions and produced an optimal policy for the robot for its interaction with the child in this game. The computed optimal policy was subsequently used for two sessions of the chase game with the same subject. Note that whereas DDQ is greedy in choosing actions during the learning process, the data obtained from the interaction with the human operator did not necessarily follow that rule, which marks a minor departure from what would have been considered a nominal DDQ implementation. Table 3 shows the accumulated rewards for all eight sessions, normalized by the time of the interaction.

**TABLE 3:** Accumulated rewards for the Dash robot. The “in” condition corresponds to the infant wearing the full-body-weight support mechanism (see Figure 6) and the “out” condition represents completely unassisted infant motion. The last two highlighted rows give outcomes on the reward obtained through the optimal policy learned by ddq. The 95*%* confidence interval for the accumulated rewards is [0.02893.4197] with a *P*-value of 0.047 7.

Session #	“In” condition	“Out” condition
1	1.081 0	5.063 2
2	2.142 8	1.836 7
3	2.043 3	1.793 4
4	2.663 5	2.468 3
5	4.258 0	5.938 5
6	3.458 6	2.544 1
7	**3.296 7**	**6.243 6**
8	**8.495 5**	**7.522 3**

To put the figures of Table 3 in proper technical context, we define a metric *I* which is a random variable that indicates the improvement as a result of using DDQ optimal policy and is expressed as $I=m_{\mathrm{DDQ}}-m_{\mathrm{human}}$ , where $m_{\mathrm{DDQ}}$ denotes the mean of the normalized accumulated rewards when the learned policy by DDQ algorithm is used (as it was in last two rehabilitation sessions), and $m_{\mathrm{human}}$ expresses the mean of the normalized accumulated rewards when the human operator decides the actions for the robot (which happened throughout the first six sessions). Here we are dealing with two small (accumulated reward) datasets that have very different standard deviations (one is more than twice of the other), and statistical comparisons necessitate the use of a *t*-test with releasing the constraint of equal standard deviation for the two group (Agresti and Finlay, 2009) in order to compute confidence interval for the random variable *I*. As it turns out, the 95*%* confidence interval is [0.0289, 3.4197] with a *P*-value of 0.0477. Since the confidence interval only includes positive numbers, and the *P*-value of the test is in an acceptable range (below 0.05), one can confidently attest that it is possible that a DDQ policy can outperform a human-driven social interaction strategy.

## 6 Conclusion

The design and implementation of an RL algorithm that captures favorable features of both model-based and model-free learning and most importantly preserves the PAC property can not only alleviate the cognitive load and time commitment of human caregivers when socially interacting in play-based activities with infants who have motor delays, but potentially also improve motor rehabilitation outcomes. One such algorithm which has been implemented and pilot-tested within an enriched robot-assisted infant motor rehabilitation environment is the DDQ. The DDQ algorithm leverages the idea of earlier Dyna-Q algorithms to combine two existing PAC algorithms, namely the model-based R-max and the model-free Delayed Q-learning, in a way that achieves the best (complexity results) of both. Theoretical analysis establishes that DDQ enjoys a sample complexity that is at worst as high as the smallest of its constituent technologies; yet, in practice, as the numerical example included suggests, DDQ can outperform them both. Numerical examples comparing DDQ to the state of the art in model-based and model free RL indicate advantages in practical implementations, and experimental implementation and testing of DDQ as it regulates a robot’s social interaction with an infant in a game of chase hints at possible advantages in rehabilitation outcomes compared to a reactive yet still goal-oriented human strategy.

## Data Availability

The original contributions presented in the study are included in the article/Supplementary Material, further inquiries can be directed to the corresponding author.
