# Supplementary material for: A Hybrid PAC Reinforcement Learning Algorithm for Human-Robot Interaction
Source: Front Robot AI. 2022 Mar 9;9:797213. doi: 10.3389/frobt.2022.797213 (PMC8982074; doi:10.3389/frobt.2022.797213)
Supplement: Supplementary file 1 [file DataSheet1.pdf]

Appendix

S1. PROOF OF LEMMA 1

Consider a fixed state-action pair (s, a). Its value $Q(s, a)$ is initially set to $v_{\max} = \frac{1}{1-\gamma}$. When an update of type-1 (Algorithm 1 line 30) is successful $Q(s, a)$ is reduced by at least ϵ_1. Since the reward function $R(s, a)$ is non-negative, we must have $Q(s, a) \geq 0$ in all timesteps, which means that there can be at most $\frac{1}{\epsilon_1(1-\gamma)}$ updates of type-1 for (s, a). On the other hand, a type-2 update (Algorithm 1 line 51) can occur only once when $n(s, a) = m_2$. Therefore, the total number of successful timesteps for (s, a) is at most $1 + \frac{1}{\epsilon_1(1-\gamma)}$ times. With $|S||A|$ total state-action pairs, the total number of successful timesteps is bounded by $\kappa = |S||A| + \frac{|S||A|}{(1-\gamma)\epsilon_1}$.

S2. PROOF OF LEMMA 2

Suppose an attempted update occurs at timestep t to some (s, a). By definition, for a subsequent attempted update to (s, a) to occur at timestep $t' > t$, at least one successful timestep must occur between t and t'. Lemma 1 ensures that there can be no more than κ successful timesteps. In other words, the most frequent occurrence of attempted updates is interlaced between successful updates, which implies that at most $1 + \kappa$ attempted updates are possible for (s, a). Scaling this argument to all state-action pairs we arrive at the $|S||A|(1 + \kappa)$ upper bound.

S3. PROOF OF LEMMA 3

Let $Q^*_{M_{K_t}}(s^*, a^*)$ denote $\max_{(s,a)} Q^*_{M_{K_t}}(s, a)$. If $(s^*, a^*) \notin K_t$, we are done since $Q^*_{M_{K_t}}(s^*, a^*) = Q_t(s^*, a^*) \leq \frac{1}{1-\gamma}$. Otherwise, for $(s^*, a^*) \in K_t$ write

$$
\begin{aligned}
Q^*_{M_{K_t}}(s^*, a^*) &= R(s^*, a^*) + \gamma \sum_s T(s^*, a^*, s) \max_a Q^*_{M_{K_t}}(s, a) \\
&\leq R(s^*, a^*) + \gamma Q^*_{M_{K_t}}(s^*, a^*) \sum_s T(s^*, a^*, s) \\
&= R(s^*, a^*) + \gamma Q^*_{M_{K_t}}(s^*, a^*) \\
&\leq 1 + \gamma Q^*_{M_{K_t}}(s^*, a^*) \implies Q^*_{M_{K_t}}(s^*, a^*) \leq \frac{1}{1-\gamma}
\end{aligned}
$$

S4. PROOF OF LEMMA 4

Let an update of type-1 occur for (s, a) at timestep t. Suppose that the latest m_1 experiences of (s, a) happened at timesteps $t_1 < t_2 < \cdots < t_{m_1} = t$, when the system was rewarded $r[1], r[2], \ldots, r[m_1]$ and jumped to states $s[1], s[2], \ldots, s[m_1]$, respectively. Define the random variable $Y = r[i] + \gamma v^*_M(s[i])$ for $1 \leq i \leq m_1$ and note that $0 \leq Y \leq \frac{1}{1-\gamma}$. Then a direct application of the Hoeffding inequality for bounded random variables and with the choice of m_1 as in (4) implies that

$$
\frac{1}{m_1} \sum_{i=1}^{m_1} \big(r[i] + \gamma v^*_M(s[i])\big) > \mathbb{E}\{Y\} - (\epsilon_1 - 2\epsilon_2) = Q^*_M(s, a) - \epsilon_1 + 2\epsilon_2
$$

with probability $1 - \delta/8(|S||A|(1+\kappa))$.

Now we have:

$$
\begin{aligned}
Q'(s,a) &= \frac{1}{m_1}\Big(\sum_{i=1}^{m_1} r[i] + \gamma v_{t_i}(s[i])\Big) + \epsilon_1 \\
&\geq \frac{1}{m_1}\Big(\sum_{i=1}^{m_1} r[i] + \gamma v_M^*(s[i])\Big) - 2\gamma\epsilon_2 + \epsilon_1 \\
&\geq Q_M^*(s,a) - \epsilon_1 + 2\epsilon_2 - 2\gamma\epsilon_2 + \epsilon_1 \geq Q_M^*(s,a)
\end{aligned}
$$

Finally, we want this fact to be true for all possible attempted updates of type-1. According to Lemma 2, an upper bound for all possible attempted updates is $|S||A|(1+\kappa)$. Therefore, the above fact is true with probability at least $\big(1 - {}^{\delta}\!/_{8(|S||A|(1+\kappa))}\big)^{|S||A|(1+\kappa)}$. An induction argument can now be employed to show that $1 - \frac{\delta}{8}$ bounds the above expression from below.

S5. PROOF OF LEMMA 7

First note that $K^2_{t_1} \subseteq K^2_{t_2}$. For all $(s,a) \notin K^2_{t_2}$

$$
Q^*_{M_{K^2_{t_1}}}(s,a) = Q_{t_1}(s,a) \geq Q_{t_2}(s,a) = Q^*_{M_{K^2_{t_2}}}(s,a) \tag{10}
$$

while for all $(s,a) \in K^2_{t_1}$

$$
Q^*_{M_{K^2_{t_1}}}(s,a) = R(s,a) + \gamma \sum_{s'} T(s,a,s') \max_{a'} Q^*_{M_{K^2_{t_1}}}(s',a')
$$

$$
Q^*_{M_{K^2_{t_2}}}(s,a) = R(s,a) + \gamma \sum_{s'} T(s,a,s') \max_{a'} Q^*_{M_{K^2_{t_2}}}(s',a')
$$

implying

$$
Q^*_{M_{K^2_{t_1}}}(s,a) - Q^*_{M_{K^2_{t_2}}}(s,a) = \gamma \sum_{s'} T(s,a,s') \times \Big(\max_{a'} Q^*_{M_{K^2_{t_1}}}(s',a') - \max_{a'} Q^*_{M_{K^2_{t_2}}}(s',a')\Big) \tag{11}
$$

Every $(s,a) \in K^2_{t_2} \setminus K^2_{t_1}$ falls in one of the following categories:

•(s,a) is a state-action pair that has not been updated ever before or at timestep t_1. The Lemma 3 implies

$$
Q^*_{M_{K^2_{t_1}}}(s,a) = Q_{t_1}(s,a) = v_{\max} = \frac{1}{1-\gamma} \geq Q^*_{M_{K^2_{t_2}}}(s,a)
$$

which completes the proof.

•(s,a) is a state-action pair that has experienced an type-1 update before or at t_1. Assume that the most recent type-1 update of (s,a) occurred at some timestep $t \leq t_1$. Suppose that the m_1 visits to (s,a) that triggered this update occurred at instances $t^1 < t^2 < \ldots < t^{m_1} = t \leq t_1$, and the observed rewards and next states were $r[1], r[2], \ldots, r[m_1]$ and $s[1], s[2], \ldots, s[m_1]$, respectively. For the random variable $Z = r[i] + \gamma v_t(s[i])$,

$$
\mathbb{E}\{Z\} = R(s,a) + \gamma \sum_{s'} T(s,a,s') \max_{a'} Q_t(s',a')
$$

Then

$$
Q^*_{M_{K^2_{t_1}}}(s,a) = Q_{t_1}(s,a) = Q_t(s,a) = \frac{\sum_{i=1}^{m_1} r[i] + \gamma v_{ti}(s[i])}{m_1} + \epsilon_1 \geq \frac{\sum_{i=1}^{m_1} r[i] + \gamma v_t(s[i])}{m_1} + \epsilon_1
$$

and applying Hoeffding inequality to the right hand side

$$
\begin{aligned}
Q^*_{M_{K^2_{t_1}}}(s,a) > \mathbb{E}\{Z\} - \epsilon_1 + 2\epsilon_2 + \epsilon_1 &= R(s,a) + \gamma \sum_{s'} T(s,a,s') \max_{a'} Q_t(s',a') + 2\epsilon_2 \\
&\geq R(s,a) + \gamma \sum_{s'} T(s,a,s') \max_{a'} Q_{t_1}(s',a') + 2\epsilon_2 \\
&\overset{(10)}{\geq} R(s,a) + \gamma \sum_{s'} T(s,a,s') \max_{a'} Q^*_{M_{K^2_{t_1}}}(s',a')
\end{aligned}
$$

with probability $1 - \frac{\delta}{8|S||A|(1+\kappa)}$. Then — following the final steps of Lemma 4 — with probability at least $1 - \frac{\delta}{8}$ after all possible attempted updates,

$$
Q^*_{M_{K^2_{t_1}}}(s,a) - Q^*_{M_{K^2_{t_2}}}(s,a) \geq \gamma \sum_{s'} T(s,a,s') \big(\max_{a'} Q^*_{M_{K^2_{t_1}}}(s',a') - \max_{a'} Q^*_{M_{K^2_{t_2}}}(s',a') \big) \tag{12}
$$

In any case, therefore, i.e., either when $(s,a) \notin K^2_{t_2}$ or when $(s,a) \in K^2_{t_2} \setminus K^2_{t_1}$, one can define

$$
\alpha := \min_{(s,a)} \big(Q^*_{M_{K^2_{t_1}}}(s,a) - Q^*_{M_{K^2_{t_2}}}(s,a) \big) := Q^*_{M_{K^2_{t_1}}}(s^*,a^*) - Q^*_{M_{K^2_{t_2}}}(s^*,a^*)
$$

and if $\alpha \geq 0$ recognize that the proof is completed. Assume for the sake of argument that $\alpha < 0$; then still either (11) is true if $(s,a) \notin K^2_{t_2}$, or (12) if $(s,a) \in K^2_{t_2} \setminus K^2_{t_1}$. Let $a_{s'} := \arg\max_{a'} Q^*_{M_{K^2_{t_2}}}(s',a')$, then in either case,

$$
\begin{aligned}
\alpha &= Q^*_{M_{K^2_{t_1}}}(s^*,a^*) - Q^*_{M_{K^2_{t_2}}}(s^*,a^*) \\
&\geq \gamma \sum_{s'} T(s^*,a^*,s') \big(\max_{a'} Q^*_{M_{K^2_{t_1}}}(s',a') - \max_{a'} Q^*_{M_{K^2_{t_2}}}(s',a') \big) \\
&= \gamma \sum_{s'} T(s^*,a^*,s') \big(\max_{a'} Q^*_{M_{K^2_{t_1}}}(s',a') - Q^*_{M_{K^2_{t_2}}}(s',a_{s'}) \big) \\
&\geq \gamma \sum_{s'} T(s^*,a^*,s') \big(Q^*_{M_{K^2_{t_1}}}(s',a_{s'}) - Q^*_{M_{K^2_{t_2}}}(s',a_{s'}) \big) \\
&\geq \gamma\alpha \implies \alpha \geq 0
\end{aligned}
$$

which is a contradiction. Therefore α cannot be negative and therefore $Q^*_{M_{K^2_{t_1}}}(s,a) - Q^*_{M_{K^2_{t_2}}}(s,a) \geq 0$.

S6. PROOF OF LEMMA 8

For all $(s,a) \notin K^2_t$

$$
Q_t(s,a) = Q^*_{M_{K^2_t}}(s,a) \leq Q^*_{M_{K^2_t}}(s,a) + 2\epsilon_2 \tag{13a}
$$

Now for $(s,a) \in K^2_t$, and referring to line 50 of Algorithm 1 one sees that for timestep t it is $Q_t(s,a) \leq Q_{\mathrm{vl}}(s,a)$. Meanwhile, for timestep t Lemma 5 ensures

$$
Q_{\mathrm{vl}}(s,a) \leq Q^*_{\hat{M}_{K^2_t}}(s,a) + \epsilon_2 \tag{13b}
$$

while Lemma 6 implies

$$
Q^*_{\hat{M}_{K^2_t}}(s,a) + \epsilon_2 \leq Q^*_{M_{K^2_t}}(s,a) + 2\epsilon_2 \tag{13c}
$$

with probability $1-\frac{\delta}{8}$. Combining (13) one obtains the right hand side of (5). Establishing the left hand side of (5) is done by strong induction. At $t=1$, we have $K_1^2=\varnothing$ and thus

$$Q_1(s,a)=Q^*_{M_{K_1^2}}(s,a)\geq Q^*_{M_{K_1^2}}(s,a)-2\epsilon_2$$

Assume that $Q_t(s,a)=Q^*_{M_{K_t^2}}(s,a)\geq Q^*_{M_{K_t^2}}(s,a)-2\epsilon_2$ for $t\leq n-1$. If timestep $t=n$ is not a successful timestep (Definition 5), nothing happens so equality holds; thus let us assume that $t=n$ is successful. Then, and for all $(s,a)\notin K_n^2$ we have automatically

$$Q_n(s,a)=Q^*_{M_{K_n^2}}(s,a)\geq Q^*_{M_{K_n^2}}(s,a)-2\epsilon_2$$

Just as before, for $(s,a)\in K_n^2$ for which a type-2 update succeeded at timestep t

$$Q_n(s,a)=Q_{\text{vl}}(s,a)\geq Q^*_{\hat{M}_{K_n^2}}(s,a)-\epsilon_2 \tag{14a}$$

as a result of Lemma 5, and

$$Q^*_{\hat{M}_{K_n^2}}(s,a)-\epsilon_2\geq Q^*_{M_{K_n^2}}(s,a)-2\epsilon_2 \tag{14b}$$

with probability $1-\frac{\delta}{8}$, due to Lemma 6. For those $(s,a)\in K_n^2$ for which a type-2 update did *not* succeed at timestep t, it is $Q_n(s,a)=Q_{n-1}(s,a)$ and there are three distinct possibilities:

- Value $Q_{n-1}(s,a)$ has never been updated before. Then,

$$Q_n(s,a)=\frac{1}{1-\gamma}\overset{\text{Lemma 3}}{\geq}Q^*_{M_{K_n^2}}(s,a)\geq Q^*_{M_{K_n^2}}(s,a)-2\epsilon_2$$

- The most recent update for (s,a) was of type-2 and occured at some $t\leq n-1$. Then,

$$Q_n(s,a)\overset{\text{Lemmas 5\&6}}{\geq}Q^*_{M_{K_t^2}}(s,a)-2\epsilon_2$$

  with probability $1-\frac{\delta}{8}$, and

$$Q^*_{M_{K_t^2}}(s,a)-2\epsilon_2\overset{\text{Lemma 7}}{\geq}Q^*_{M_{K_n^2}}(s,a)-2\epsilon_2$$

  also with with probability $1-\frac{\delta}{8}$, so

$$Q_n(s,a)\geq Q^*_{M_{K_n^2}}(s,a)-2\epsilon_2$$

  with probability at least $1-\frac{2\delta}{8}\leq(1-\frac{\delta}{8})^2$.
- The most recent update for (s,a) was of type-1 and occured at some $t'\leq n-1$. Then suppose that the m_1 collection of visits of (s,a) for this update occurred at timesteps $t^1<t^2<\cdots<t^{m_1}=t^{'}\leq n-1$, with the corresponding observed reward and next states being $r[1],r[2],\ldots,r[m_1]$ and $s[1],s[2],\ldots,s[m_1]$, respectively. The expectation of the random variable $F=r[i]+\gamma v_{t^{m_1}}(s[i])$ is

$$\mathbb{E}\{F\}=R(s,a)+\gamma\sum_{s'}T(s,a,s')\max_{a'}Q_{t^{m_1}}(s',a')$$

which, with the use of Hoeffding inequality, bounds the sum in

$$\begin{aligned} Q_n(s,a) &= \frac{1}{m_1}\left(\sum_{i=1}^{m_1} r[i] + \gamma v_{t^i}(s[i])\right) + \epsilon_1 \\ &\geq \frac{1}{m_1}\left(\sum_{i=1}^{m_1} r[i] + \gamma v_{t^{m_1}}(s[i])\right) + \epsilon_1 > \mathbb{E}\{F\} - \epsilon_1 + 2\epsilon_2 + \epsilon_1 \\ &= R(s,a) + \gamma\sum_{s'} T(s,a,s')\max_{a'} Q_{t'}(s',a') + 2\epsilon_2 \end{aligned}$$

and yields

$$Q_n(s,a) \geq R(s,a) + \gamma\sum_{s'} T(s,a,s')\max_{a'} Q_n(s',a')$$

with probability $1 - \delta/8(|S||A|(1+\kappa))$. Following the steps in the proof of Lemma 4 when thinking of all possible attempted updates, one states the above with probability $1 - \frac{\delta}{8}$. Subtracting now $Q^*_{M_{K_n^2}}(s,a)$ from both sides yields

$$\gamma\sum_{s'} T(s,a,s')\left(\max_{a'} Q_n(s',a') - \max_{a'} Q^*_{M_{K_n^2}}(s',a')\right) \leq Q_n(s,a) - Q^*_{M_{K_n^2}}(s,a) \tag{15}$$

and if one denotes

$$\alpha := \min_{(s,a)}\left(Q_n(s,a) - Q^*_{M_{K_n^2}}(s,a)\right) = Q_n(s^*,a^*) - Q^*_{M_{K_n^2}}(s^*,a^*)$$

then we want to show $\alpha \geq -2\epsilon_2$. Let $a_{s'} := \arg\max_{a'} Q^*_{M_{K_n^2}}(s',a')$, then (15) implies

$$\begin{aligned} \alpha &= Q_n(s^*,a^*) - Q^*_{M_{K_n^2}}(s^*,a^*) \\ &\geq \gamma\sum_{s'} T(s^*,a^*,s')\left(\max_{a'} Q_n(s',a') - \max_{a'} Q^*_{M_{K_n^2}}(s',a')\right) \\ &= \gamma\sum_{s'} T(s^*,a^*,s')\left(\max_{a'} Q_n(s',a') - Q^*_{M_{K_n^2}}(s',a_{s'})\right) \\ &\geq \gamma\sum_{s'} T(s^*,a^*,s')\left(Q_n(s',a_{s'}) - Q^*_{M_{K_n^2}}(s',a_{s'})\right) \\ &\geq \gamma\alpha \implies \alpha \geq 0 \geq -2\epsilon_2 \end{aligned}$$

Summing up, the right side of (5) holds with probability $1 - \frac{\delta}{8}$, while the left side is true with probability at least $(1 - \frac{\delta}{12})^2$. Together, both inequalities are true with probability at least $(1 - \frac{\delta}{12})^3 \geq 1 - \frac{3\delta}{8}$.

S7. PROOF OF LEMMA 9

Assume that at timestep t, $(s,a) \notin K_t$, $l(s,a) = 0$ and $\mathsf{learn}(s,a) = \text{true}$, and suppose that m_1 experiences of (s,a) after t happen at timesteps $t \leq t_1 < t_2 < \cdots < t_{m_1}$. Let $r[1], r[2], \ldots, r[m_1]$ and $s[1], s[2], \ldots, s[m_1]$ be the rewards and next states observed for the m_1 experiences of (s,a). Then define the random variable $X = r[i] + \gamma v_{t_1}(s[i])$ letting i range in $\{1, \ldots, m_1\}$, and note that $0 \leq X \leq 1$.

A direct application of the Hoeffding inequality with the choice of m_1 as in (4) yields

$$\frac{1}{m_1}\left(\sum_{i=1}^{m_1} r[i] + \gamma v_{t_1}(s[i])\right) - \mathbb{E}\{X\} < \epsilon_1 - 2\epsilon_2 < \epsilon_1$$

with probability $1 - \frac{\delta}{8|S||A|(1+\kappa)}$. Since the DDQ algorithm only allows for updates that decrease the value estimate for any stat-action pairs, we can write:

$$Q_t(s,a) - \frac{1}{m_1}\left(\sum_{i=1}^{m_1} r[i] + \gamma v_{t_i}(s[i])\right) \geq Q_t(s,a) - \frac{1}{m_1}\left(\sum_{i=1}^{m_1} r[i] + \gamma v_{t_1}(s[i])\right)$$
$$> Q_t(s,a) - \mathbb{E}\{X\} - \epsilon_1$$

and because $(s,a) \notin K_t$ meaning $Q_t(s,a) - \mathbb{E}\{X\} > 3\epsilon_1$,

$$Q_t(s,a) - \mathbb{E}\{X\} - \epsilon_1 > 2\epsilon_1$$

guaranteeing success for the type-1 update at timestep t_{m_1}. Since for the case that $l(s,a) = 0$ and $\mathsf{learn}(s,a) = \mathrm{true}$, an attempted update will necessarily happen; there can be at most $|S||A|(1+\kappa)$ instances of such an event. Working in a fashion similar to the proof of Lemma 4, one concludes that the lemma's statement holds with probability at least $1 - \frac{\delta}{8}$.

S8. PROOF OF LEMMA 10

We will assume that (s,a) has not already been visited m_2 times before timestep t, because then it is obvious that $(s,a) \in K_{t+1}$. Thus we work under the assumption that (s,a) has been visited fewer than m_2 times up until t, at which time an unsuccessful update of (s,a) occurs, while right after at $t+1$ we see $\mathsf{learn}(s,a) = \mathrm{false}$. Now set up a contradiction argument: under those conditions, *assume that* $(s,a) \notin K_{t+1}$. Since the update at t was unsuccessful, $K_{t+1} = K_t$, which would also imply that $(s,a) \notin K_t$. Now label the times of the most recent m_1 experiences of (s,a) as $b(s,a) \triangleq t_1 < t_2 < \cdots < t_{m_1} = t$. The contrapositive of the statement proved in Lemma 9, suggests that since the update at t is unsuccessful, it must be $(s,a) \in K_{t_1}$. Since $(s,a) \notin K_t$, some timestep between t_1 and t must have been successful. Let us denote that successful timestep $t^* > b(s,a)$. But then the condition $t_1 = b(s,a) < t^*$ would not allow the learn flag to be set to false in between these two timesteps, and we know from the statement of the lemma that this is true. Therefore, we have a contradiction; the assumption made is invalid, and therefore $(s,a) \in K_t = K_{t+1}$.

S9. PROOF OF LEMMA 11

Fix a state-action pair (s,a), We begin by showing that if $(s,a) \notin K_t$ at timestep t, then within at most $2m_1$ more experiences of (s,a) after t, a successful timestep for (s,a) must occur. Toward that end, we analyse the worst case where m_2-th visit of (s,a) will not occur within $2m_1$ more experiences of (s,a) after timestep t. For $(s,a) \notin K_t$, distinguish two possible cases at the beginning of timestep t: either $\mathsf{learn}(s,a) = \mathrm{false}$ or $\mathsf{learn}(s,a) = \mathrm{true}$. Consider first the case where $\mathsf{learn}(s,a) = \mathrm{false}$. Assume that the most recent attempted update of (s,a) occurred at some timestep t' which was unsuccessful and set the flag $\mathsf{learn}(s,a)$ to false. Then, according to Lemma 10, it will be $(s,a) \in K_{t'+1}$. However, now it is $(s,a) \notin K_t$, which implies that a successful timestep must have occurred at some t^* with $t'+1 < t^* < t$. Thus the flag $\mathsf{learn}(s,a)$ will set to true during timestep t. Then, at t we have all conditions of Lemma 9 (i.e. $\mathsf{learn}(s,a) = \mathrm{true}$, $(s,a) \notin K_t$ and $l(s,a) = 0$) and thus the type-1 update upon m_1-th visit of (s,a) after t will be successful.

Take now the case where $\mathsf{learn}(s,a) = \mathrm{true}$. We know that an attempted type-1 update for (s,a) will occur in at most m_1 experiences of (s,a), and those are assumed occurring at timesteps $t_1 < \cdots < t_{m_1}$, then $t_1 \leq t \leq t_{m_1}$. Consider the two possibilities: $(s,a) \notin K_{t_1}$ or $(s,a) \in K_{t_1}$. In the former case, Lemma 9 indicates that the attempted update type-1 at t_{m_1} will be successful. In the latter case, given that $(s,a) \notin K_t$, a successful timestep t^* must have taken place between t_1 and t (since $K_{t_1} \neq K_t$). Thus, however the attempted update at t_{m_1} is unsuccessful, $\mathsf{learn}(s,a)$ will remain true and at timestep $t_{m_1}+1$ we will have $\mathsf{learn}(s,a) = \mathrm{true}$, $l(s,a) = 0$, and $(s,a) \notin K_{t_{m_1}+1}$; this would trigger Lemma 9, and the attempted update type-1 upon m_1-th visit of (s,a) after timestep $t_{m_1}+1$ (which is within at most $2m_1$ more experiences of (s,a) after t), will be successful.

Thus far, we showed that after $(s,a) \notin K_t$, within at most $2m_1$ more experiences of (s,a), at least one successful timestep for (s,a) must occur. According to lemma 1, the total number of successful timesteps

for (s, a) are bounded by $1 + \frac{1}{(1-\gamma)\epsilon_1}$. This means that the total number of timesteps with $(s, a) \notin K_t$ is bounded by $2m_1(1 + \frac{1}{(1-\gamma)\epsilon_1})$. On the other hand, once a state-action pair (s, a) is experienced for m_2-th time at any timestep t, it will become a member of K_t and will never leave K_t anymore. So, m_2 is another upper-bound for the number of timesteps with $(s, a) \notin K_t$.

Generalizing the above fact for all state-action pairs, we conclude that the total number of escape events (timesteps t with $(s_t, a_t) \notin K_t$) is bounded by $\min(2m_1\kappa, |S||A|m_2)$.
